# Influence of Vitamin K on Bone Mineral Density and Osteoporosis

**DOI:** 10.7759/cureus.10816

**Published:** 2020-10-05

**Authors:** Abeer O Elshaikh, Lisa Shah, Christopher Joy Mathew, Robert Lee, Merin Tresa Jose, Ivan Cancarevic

**Affiliations:** 1 Internal Medicine, California Institute of Behavioral Neurosciences & Psychology, Fairfield, USA; 2 Family and Community Medicine, California Institute of Behavioral Neurosciences & Psychology, Fairfield, USA; 3 Medicine, California Institute of Behavioral Neurosciences & Psychology, Fairfield, USA; 4 Surgery, California Institute of Behavioral Neurosciences & Psychology, Fairfield, USA; 5 Family Medicine, California Institute of Behavioral Neurosciences & Psychology, Fairfield, USA

**Keywords:** vitamin k, osteoporosis, vitamin k-dependent protein

## Abstract

Vitamin K (VK) has an established biological function in blood coagulation and hemostasis and maintains general health and bone wellbeing. VK supplements have been promoted to treat and prevent many diseases, particularly for decreasing fracture risk in osteoporosis, a chronic condition described by weak bone tissue, and a high fracture risk following minor trauma. It affects older people from different races and ethnicity, mainly postmenopausal women. Many kinds of research emphasize the role of VK in improving bone health and preventing osteoporotic bone fracture, but the findings are mostly inconclusive. In this literature review, PubMed and Google Scholar databases were used as the primary sources to select the relevant studies and review the association between VK and bone health and also, to explore the impact of VK supplementation in osteoporosis management. A majority of studies reported that VK has an essential role in promoting bone health. Although some studies revealed that VK might increase bone mineral density and reduce fracture risk in people with osteoporosis, VK supplements' potential benefits were not sufficiently supported. Thus, more clinical studies are needed to determine the positive effects of VK supplementation in osteoporosis prevention and treatment.

## Introduction and background

Vitamin K (VK) is a lipid-soluble vitamin that contributes to blood coagulation and maintenance of bone health [1]. There are three known types of VK: phylloquinone (PK), menaquinone (MK), and menadione, which are called vitamins K1, K2, K3, respectively [2]. PK is a dietary form of vitamin K and is created by plants and green vegetables like spinach and kale. MKs are synthesized by intestinal normal flora and can also be found in fermented food like natto (fermented soybeans), dairy items, egg yolk, liver, and meat. In the presence of a functioning pancreas and biliary system, vitamins K1 and K2 are absorbed in the small and large intestine [3]. They are subsequently transported in triglyceride-rich chylomicrons in the lymphatic system. MKs are categorized according to the length of their unsaturated side chains. There are 12 different types of MKs (MK-4 to MK-15). The most common types of MKs in humans are short-chain MK-4, which is produced by the conversion of phylloquinone to menaquinones, and long-chain vitamers, MK-7 to MK-10, which are synthesized by bacteria in the human body.

Vitamin K impacts bones in different ways [4]. It is an essential coenzyme for the gamma-glutamyl carboxylase enzyme reaction, which converts glutamic acid (Glu) residues in VK-dependent proteins (VKDPs) to gamma-carboxyglutamic acid (GIa). There are many VKDPs in the bone, including osteocalcin (OC), matrix Gla protein (MGP), gas 6, periostin, and protein S [3]. Vitamin K also regulates the transcription of osteoblastic markers, the formation of osteoclasts, and bone resorption. Many studies found that low serum vitamin K1 concentrations are associated with high levels of undercarboxylated osteocalcin (ucOC) [3]. The low dietary intake of vitamin K1 and vitamin K2 increases the risk of fracture.

Osteoporosis is a chronic disease characterizes by weak bone tissue, which leads to a significant increase in the risk of bone fragility and fracture [5]. The prevalence of osteoporosis-related fractures increases rapidly with age, from 4% in women at age 50-59 to 52% in women aged >80 years [6]. Many risk factors are linked to osteoporosis development like age, gender, ethnicity, genetic factors, reproductive status, low calcium intake, lifestyle, and certain diseases [7]. The recommended guideline of the United States Preventive Services Task Force (USPSTF) is the dual-energy x-ray absorptiometry (DXA) screening in women aged 65 years and older, women less than 65 years of age with one or multiple risk factors, and women with a history of fractures [6]. Depending on the bone mineral density (BMD) test results and a fracture risk assessment, pharmacological and non-pharmacological interventions are employed to treat osteoporosis. The treatment objectives are the prevention of bone fractures, maintaining BMD, and improving physical function. Calcium and vitamin D have been viewed as a foundation in the treatment of postmenopausal osteoporosis [7]. Currently, all affected individuals should take 1000-1200 mg of calcium and 800 IU of vitamin D as supplementation.

This literature review objective is to discuss the impact of vitamin K on bone health and investigate the association between vitamin K supplements and osteoporosis treatment and prevention. PubMed and Google Scholar databases were used as the primary sources for this review study to select the relevant papers. Selected papers have been published within the last 20 years and written in the English language. Observational studies, randomized clinical trials, in vitro and animal studies were also included.

## Review

Vitamin K-dependent proteins and vitamin K status

**Figure 1:**
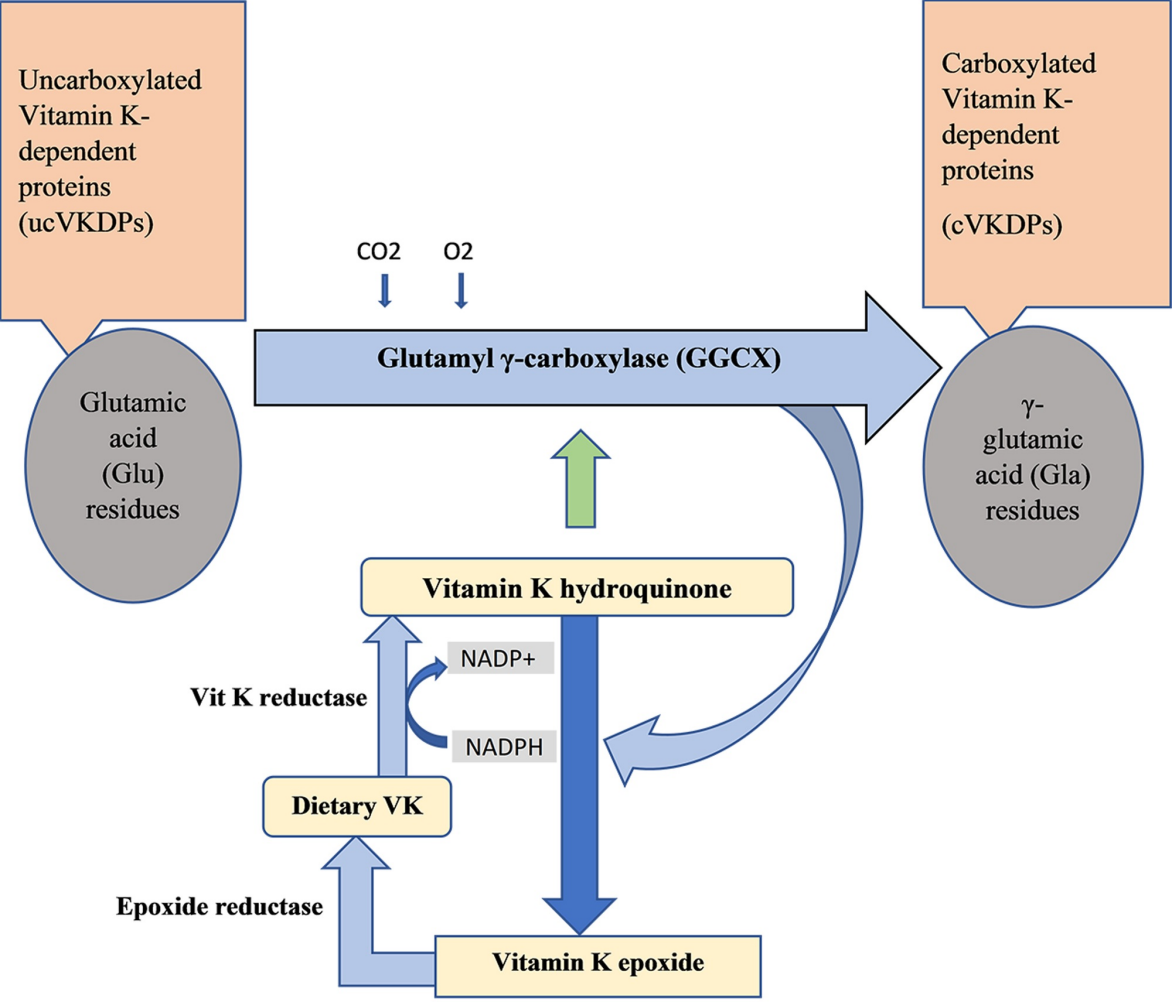
Carboxylation of vitamin K dependent proteins NADP: nicotinamide adenine dinucleotide phosphate; NADPH: nicotinamide adenine dinucleotide phosphate hydrogen

VKDPs require carboxylation of specific glutamate residues as the reaction elevates their affinity for calcium [8]. Therefore, dietary vitamin K is converted to its reduced form (hydroquinone) by vitamin K reductase; after carboxylation, vitamin K epoxide reductase converts vitamin K epoxide to vitamin K (dietary vitamin k) [9]. The reactions are illustrated in Figure 1. Vitamin K works as a coenzyme for the glutamate γ-carboxylase (GGCX) enzyme and is needed to convert VKDP glutamic acid residues to γ-glutamic acid residues [9]. Seven kinds of VKDPs are involved in blood coagulation and hemostasis, including coagulation factor prothrombin (factor II), proconvertin (factor VII), antihemophilic (factor IX), and Stuart-Prower factor (factor X). There are four VKDPs found in bone from the transmembrane Gla family [10]. The other VKDPs include MGP, growth arrest-specific protein 6 (Gas6), and protein S. OC contains 49 amino acid residues and is synthesized and secreted by osteoblasts, odontoblasts, and hypertrophic cartilage cells [9]. The OC gene of humans is located on chromosome one. MGP is synthesized by chondrocytes, osteoclasts, and vascular smooth muscle cells; the protein promotes normal bone metabolism [10]. Gla-rich protein and periostin regulate extracellular matrix mineralization; protein S is mainly synthesized in the liver and plays a major role in the anticoagulation pathway, but it is also secreted by osteoblasts and involved in the bone turnover.

Direct and indirect methods are used to measure vitamin K levels in the body and determine deficiency. Common direct methods include quantifying urinary vitamin K1 (phylloquinone) metabolites and circulating forms of vitamin K1 and K2 (menaquinone) [9]. A considerable number of clinical studies describe the use of indirect methods such as measuring circulating markers that represent ucOC to reflect extra-hepatic vitamin K status or studying the effects of vitamin K supplementation and its association with a particular health condition [9]. The ucOC level is vitamin K dependent and considered an indirect biomarker of vitamin K status. The circulating OC is used as a biomarker for bone formation [10]. Regardless of plasma vitamin K concentration, OC is correlated to bone turnover and metabolism. The level of prothrombin time (PT) is measured to assess the coagulation function and may reflect VK deficiency; the deficiency is rare and mostly due to the intake of specific medications like VK antagonist anticoagulants, antibiotics, anticonvulsants, and those for liver disease and pancreas disease [10].

Molecular action of vitamin K on bone tissue

Vitamin K has an essential role in bone health and functions through different mechanisms. OC binds to calcium ions and hydroxyapatite crystals, regulating their shape and size [4]. The transcription and translation of the OC gene are controlled and regulated by vitamin D 1,25(OH)2 D3, but its ability to link with calcium ions depends on vitamin K [10]. MGP facilitates normal bone metabolism, and it has been found that MGP attains optimal biological activity just after post-translational carboxylation. Gla-rich protein and periostin regulate extracellular matrix mineralization [4]. Also, VK can regulate gene transcription of osteoblastic markers, suppress bone resorption, and regulate osteoclasts' formation. Animal and in vitro studies showed that menaquinone (MK-4) might be involved in inflammation, apoptosis, and oxidative stress all of which can inhibit bone resorption [4]. MK-4 acts through steroid and xenobiotic receptor (SXR) to regulate the expression of different genes that control the osteoblast formation and differentiation [11]. Tabb et al. found that vitamin K2 ( menaquinone) activated SXR in a dose-dependent process and regulated the expression of osteoblast marker genes that promote bone deposition and decrease bone resorption [12]. Interestingly, SXR is also expressed in osteosarcoma cell lines that are considered as osteoblastic cells in origin and function as both a mediator in bone homeostasis and a xenobiotic sensor. Ichikawa et al. performed a study to identify the SXR target genes and found that tsukushi, matrilin-2, and CD14 antigen are primary SXR target genes, and all three genes have bone-related functions [13]. For example, collagen accumulation in osteoblastic cells was enhanced by vitamin K2 treatment and tsukushi, a small leucine-rich proteoglycan, contributes to this process, as demonstrated by the gain of function and loss of function analyses. Their results suggest a new function for vitamin K2 in bone formation as a transcriptional regulator of extracellular matrix-related genes involved in the collagen assembly. Koshihara et al. demonstrated that vitamin K1 (phylloquinone) and K2 administration decreased the expression of receptor activator of nuclear factor kappa B ligand (RANKL) and increased the expression of osteoclastogenesis-inhibitory factor also known as osteoprotegerin (OPG) in the stromal cells [14]. They concluded that vitamin K might stimulate osteoblast genesis in bone marrow cells.

Several studies investigated the biological action of vitamin K on bone tissue. Most of them stated that the essential role of vitamin K in osteoblastic function is mediated through the classical protein γ-carboxylation pathway, which is well established. However, other studies explored different mechanisms of vitamin K action on bone and showed evidence of osteoprotective action of vitamin K2 is mediated through the upregulating of bone marker genes. Targeting these genes may be utilized as a new therapeutic agent for treating several bone diseases, including osteoporosis. The actions of vitamin K on bone are summarized in Table 1.

**Table 1:** Summary of vitamin K actions on bone tissue GGCX: glutamate γ-carboxylase; Gla: gamma-carboxyglutamic acid; RANKL: receptor activator of nuclear factor kappa B ligand; SXR: steroid and xenobiotic receptor; matrix Gla protein

Vitamin K actions		
Coenzyme for the GGCX [9]	Suppress bone resorption [4]	Increase osteoblast genesis [11]
Gla osteocalcin formation [9]	Regulate osteoclast formation [4]	Enhance osteoblast collagen accumulation [13]
Gla-rich protein and periostin production [4]	Decrease RANKL expression [14]	Increase the expression of osteoprotegerin (OPG) [14]
MGP maintains bone metabolism [10]	Induce osteoclast apoptosis [4]	SXR signaling induce osteoblast differentiation [11]

Effect of vitamin K on BMD

Multiple studies were conducted to evaluate the effect of vitamin K on BMD. The majority of them showed that adequate VK intake has indeed improved BMD [15]. Women who had the lowest vitamin K1 (phylloquinone) intake level had a significantly lower mean BMD after adjusting for confounding factors like menopausal status and age. Some contrasting studies show no significant association between VK intake and BMD. Huang et al. performed a meta-analysis of 19 randomized, double-blind, placebo-controlled trials, which included 6759 participants, to investigate the effects that a daily intake of 1.5 mg short-chain menaquinone-4 (MK-4) has on BMD [16]. They found significant improvement of vertebral BMD in postmenopausal women with osteoporosis but no significant changes in BMD were reported for postmenopausal women without osteoporosis. These results suggest that low-dose MK-4 supplementation for 6-12 months improved bone quality in the postmenopausal Japanese women without any substantial adverse effects by decreasing the serum ucOC and pentosidine concentrations. Knapen et al. studied healthy postmenopausal women who received either placebo or long-chain menaquinone-7 (MK-7) 180 mcg/day capsules for three years [17]. During the first year, the rate of bone loss was similar in both groups, but after three years, MK-7 positively affected bone health compared to placebo even after adjusting for age and body mass index (BMI). Additional analysis showed no differences in femoral neck width, hip-axis length, and bending strength but slight differences in terms of compression strength and a significant difference in age-adjusted impact strength. Moschonis et al. performed a randomized controlled trial (RCT) on postmenopausal women who were given fortified dairy products plus vitamin K1 100 mcg, vitamin K2 (menaquinone) 100 mcg, or a placebo (regular diet) [18]. After 12 months, the intervention groups showed a significant increase in total BMD compared to the control. Emaus et al. conducted a randomized, double-blind, placebo-controlled trial on postmenopausal women to study the effects of consuming 1.5 mg of MK-4 daily on various bone turnover markers (BTMs) and BMD [19]. The results showed no significant impact after one year of natto (fermented soybeans) on BMD in healthy postmenopausal 50- to 60-year-old Norwegian women. However, serum levels of carboxylated osteocalcin (cOC) increased, and levels of ucOC decreased in the treatment versus the placebo group (p < 0.001). Binkley et al. conducted an RCT on postmenopausal women receiving calcium and cholecalciferol (VD3) plus VK1 (1 mg daily), MK-4 (45 mg daily), or placebo for 12 months [20]. They found no significant difference in lumbar or hip BMD between the two groups. Cheung et al. performed an RCT on postmenopausal women with osteopenia and found no significant differences in BMD at any site between the two groups over the two- to four-year period [21]. Daily vitamin K1 supplementation increased serum vitamin K1 levels by 10-fold.

Several studies were performed to investigate the effects of VK on BMD and bone remodeling biomarkers. All these studies included cross-sectional studies and RCTs in different populations. Most studies suggested a positive effect of VK on BMD, a higher level of cOC, and a low level of ucOC [16,19]. On the other hand, some studies showed no significant association between VK intake and BMD [19-21]. It seems that many factors and cofounders play a role in the contradicting results like different population samples, age, diet, and health status. We need more studies to confirm the association between VK and bone biomarkers as well as BMD.

Effect of vitamin K on osteoporosis and bone fracture

Several RCTs and cross-sectional studies were performed to investigate the effect of VK on bone fracture prevention. Knapen et al. investigated the impact of low-dose vitamin K2 (menaquinone) supplement MK-7 (long-chain menaquinone-7) 180 mcg/day on bone health [17]. The researchers performed DXA to assess vertebral fractures in healthy postmenopausal women for three years compared to placebo. They reported that after two and three years, the vertebrae's height loss was significantly lower in the MK-7 group than in the placebo group. Cheung et al. performed a four-year study on postmenopausal women with osteopenia and normal levels of vitamin D [21]. They found that fewer women on vitamin K1 (phylloquinone) 500 mcg/day had a clinical fracture than the placebo. Nakano et al. performed a case-control study in elderly Asian patients with hip fracture versus controls [22]. They concluded that a low concentration of vitamin K1 was associated with an increased risk of fracture. Kasukawa et al. conducted a study to explore the effect of vitamin K2 in addition to risedronate in an RCT [23]. The study consisted of 101 older women with postmenopausal osteoporosis, and the researchers found no significant difference in terms of vertebral fracture incidence. Chan et al. performed a cross-sectional study on elderly Asian men and women over 65 years old [24]. The study showed that VK intake was not correlated with fracture risk in either sex after almost seven years of follow-up, even after adjustment for confounding factors. Rejnmark et al. performed a study within the Danish Osteoporosis Prevention Study (DOPS) [25]. It included a population-based cohort of 2,016 perimenopausal women over 10 years to investigate the association between vitamin K1 intake and BMD as well as fracture risk. They reported no effect on BMD and risk of fracture in perimenopausal women. Huang et al. found that vitamin K2 treatment improved vertebral BMD and reduced fracture risk in postmenopausal women with osteoporosis [16]. However, no effect appeared in postmenopausal women without osteoporosis. Tusar et al. recently conducted a nine-week, prospective cohort, an open-labeled study in 29 postmenopausal women who suffered a vertebral or hip osteoporotic compression fracture, to investigate the improvement in the carboxylation of OC in response to escalating doses of MK-4 supplementation [26]. They reported that in postmenopausal women with osteoporotic fractures, either 5 or 45 mg/day MK-4 supplementation resulted in marginal increases in γ-carboxylated osteocalcin and decreased ucOC to concentration usually found in premenopausal healthy women.

Many available studies, including a few RCTs performed to investigate the effects of vitamin K1 (phylloquinone) and vitamin K2 (menaquinone) on fracture risk, showed a potential relationship between the low concentration of VK and the increased risk of fracture in different populations [22]. However, many other studies reported no effect of vitamin K supplements on fracture risk [23-25]. This contradicts findings appearing in many observational studies. It seems that the positive effect was more common in healthy people. Further studies are needed to confirm the effects of vitamin K supplementation on reducing the risk of bone fracture. In the context of the impact of vitamin K on women with osteoporosis, the available studies did not report clear benefits, and more researches are needed to verify the positive outcome of vitamin K supplements in individuals with osteoporosis.

## Conclusions

Several published studies indicate that vitamin K has a positive effect on bone health. It seems that vitamin K enhances BMD and increases the level of cOC. The latter of the two effects is inversely related to bone deterioration and fracture. However, the benefit of vitamin K supplementation for osteoporosis prevention and treatment is still controversial. Some studies showed decreased osteoporosis-related fractures after vitamin K supplementation, whereas others did not get the same results. We need additional clinical research before making a recommendation about vitamin K supplementation for the treatment and prevention of osteoporosis. These researches should be conducted in a large population and contain more RCTs to prove the benefits of vitamin K supplementation.

## References

[1] Evenepoel P, Claes K, Meijers B (2019). Poor vitamin K status is associated with low bone mineral density and increased fracture risk in end-stage renal disease. J Bone Miner Res.

[2] Klapkova E, Cepova J, Dunovska K, Prusa R (2018). Determination of vitamins K1 , MK-4, and MK-7 in human serum of postmenopausal women by HPLC with fluorescence detection. J Clin Lab Anal.

[3] Fusaro M, Gallieni M, Rizzo M (2017). Vitamin K plasma levels determination in human health. Clin Chem Lab Med.

[4] Rodríguez-Olleros Rodríguez C, Díaz Curiel M (2019). Vitamin K and bone health: a review on the effects of vitamin K deficiency and supplementation and the effect of non-vitamin K antagonist oral anticoagulants on different bone parameters. J Osteoporos.

[5] Baccaro LF, Conde DM, Costa-Paiva L, Pinto-Neto AM (2015). The epidemiology and management of postmenopausal osteoporosis: a viewpoint from Brazil. Clin Interv Aging.

[6] Tella SH, Gallagher JC (2014). Prevention and treatment of postmenopausal osteoporosis. J Steroid Biochem Mol Biol.

[7] Eriksen EF, Halse J, Moen MH (2013). New developments in the treatment of osteoporosis. Acta Obstet Gynecol Scand.

[8] Wasilewski GB, Vervloet MG, Schurgers LJ (2019). The bone-vasculature axis: calcium supplementation and the role of vitamin K. Front Cardiovasc Med.

[9] Wen L, Chen J, Duan L, Li S (2018). Vitamin K‑dependent proteins involved in bone and cardiovascular health (review). Mol Med Rep.

[10] Akbari S, Rasouli-Ghahroudi AA (2018). Vitamin K and bone metabolism: a review of the latest evidence in preclinical studies. Biomed Res Int.

[11] Hirota Y, Suhara Y (2019). New aspects of vitamin K research with synthetic ligands: transcriptional activity via SXR and neural differentiation activity. Int J Mol Sci.

[12] Tabb MM, Sun A, Zhou C (2003). Vitamin K2 regulation of bone homeostasis is mediated by the steroid and xenobiotic receptor SXR. J Biol Chem.

[13] Ichikawa T, Horie-Inoue K, Ikeda K (2006). Steroid and xenobiotic receptor SXR mediates vitamin K2-activated transcription of extracellular matrix-related genes and collagen accumulation in osteoblastic cells.. J Biol Chem.

[14] Koshihara Y, Hoshi K, Okawara R (2003). Vitamin K stimulates osteoblastogenesis and inhibits osteoclastogenesis in human bone marrow cell culture. J Endocrinol.

[15] Akbari S, Rasouli-Ghahroudi AA (2018). Vitamin K and bone metabolism: a review of the latest evidence in preclinical studies. Biomed Res Int.

[16] Huang ZB, Wan SL, Lu YJ, Ning L, Liu C, Fan SW (2015). Does vitamin K2 play a role in the prevention and treatment of osteoporosis for postmenopausal women: a meta-analysis of randomized controlled trials. Osteoporos Int.

[17] Knapen MHJ, Drummen NE, Smit E, Vermeer C, Theuwissen E (2013). Three-year low-dose menaquinone-7 supplementation helps decrease bone loss in healthy postmenopausal women. Osteoporos Int.

[18] Moschonis G, Kanellakis S, Papaioannou N, Schaafsma A, Manios Y (2011). Possible site-specific effect of an intervention combining nutrition and lifestyle counselling with consumption of fortified dairy products on bone mass. J Bone Miner Metab.

[19] Emaus N, Gjesdal CG, Almås B (2010). Vitamin K2 supplementation does not influence bone loss in early menopausal women: a randomised double-blind placebo-controlled trial. Osteoporos Int.

[20] Binkley N, Harke J, Krueger D (2009). Vitamin K treatment reduces undercarboxylated osteocalcin but does not alter bone turnover, density, or geometry in healthy postmenopausal North American women. J Bone Miner Res.

[21] Cheung AM, Tile L, Lee Y (2008). Vitamin K supplementation in postmenopausal women with osteopenia (ECKO trial): a randomized controlled trial. PLoS Med.

[22] Nakano T, Tsugawa N, Kuwabara A, Kamao M, Tanaka K, Okano T (2011). High prevalence of hypovitaminosis D and K in patients with hip fracture. Asia Pac J Clin Nutr.

[23] Kasukawa Y, Miyakoshi N, Ebina T (2014). Effects of risedronate alone or combined with vitamin K2 on serum undercarboxylated osteocalcin and osteocalcin levels in postmenopausal osteoporosis. J Bone Miner Metab.

[24] Chan R, Leung J, Woo J (2012). No association between dietary vitamin K intake and fracture risk in chinese community-dwelling older men and women: a prospective study. Calcif Tissue Int.

[25] Rejnmark L, Vestergaard P, Charles P, Hermann AP, Brot C, Eiken P, Mosekilde L (2006). No effect of vitamin K1 intake on bone mineral density and fracture risk in perimenopausal women. Osteoporos Int.

[26] Giri TK, Newton D, Chaudhary O (2020). Maximal dose-response of vitamin-K2 (menaquinone-4) on undercarboxylated osteocalcin in women with osteoporosis. Int J Vitam Nutr Res.

